# Sparse PrP^Sc ^accumulation in the placentas of goats with naturally acquired scrapie

**DOI:** 10.1186/1746-6148-7-7

**Published:** 2011-02-01

**Authors:** Katherine I O'Rourke, Dongyue Zhuang, Thomas C Truscott, Huijan Yan, David A Schneider

**Affiliations:** 1 Animal Disease Research Unit, Agricultural Research Service, U.S. Department of Agriculture, Pullman WA 99164 USA; 2 Department of Veterinary Microbiology and Pathology, College of Veterinary Medicine, Washington State University, Pullman WA 99164 USA

## Abstract

**Background:**

Domestic goats (*Capra hircus*) are a natural and experimental host of scrapie and bovine spongiform encephalopathy, the transmissible spongiform encephalopathies (TSE) of sheep and cattle. Goats are also susceptible to experimental infection with the agents of TSEs of deer and elk (chronic wasting disease) and humans (Creutzfeldt Jakob disease). Distribution of PrP^Sc^, the abnormal prion protein, is similar in the tissues of scrapie-infected sheep and goats but no data are available on the potential shedding of the agent through the placenta, the presumed route of transmission of ovine scrapie. We describe the sparse accumulation of PrP^Sc ^in the placentas of goats with naturally acquired classical scrapie in comparison to field cases of classical ovine scrapie.

**Results:**

PrP^Sc ^was detected in the shed placentas from a sample of U.S. goats with naturally occurring scrapie, diagnosed by antemortem lymphoid tissue biopsy or identified as high risk progeny of infected dams. PrP^Sc ^accumulation patterns in the intact placentome and western blot banding was similar in the caprine and ovine samples. However, levels of PrP^Sc ^estimated from ELISA and immunohistochemistry assays were generally lower in goats than in sheep, although wide variation was noted in both species.

**Conclusions:**

PrP^Sc ^accumulates in the shed placentas of goats with naturally acquired scrapie. Although these levels were low in most caprine samples, the caprine placenta may contribute to prion contamination of kidding facilities and transmission to co-housed sheep or goats.

## Background

Scrapie, a neurodegenerative disease of domestic sheep (*Ovis aries*), is the prototype transmissible spongiform encephalopathy (TSE) of ruminant livestock. The ruminant TSEs are a family of diverse infectious and possibly sporadic disorders of sheep, cattle, deer, elk, moose, and goats. Classical studies in rodent models have demonstrated that the infectious agent is composed primarily of a relatively protease resistant isoform (PrP^Sc^) of the highly conserved mammalian prion protein (PrP^c^) encoded by the PRNP gene [1]. Although PrP^c ^is a ubiquitously expressed membrane sialoglycoprotein [2], PrP^Sc ^accumulates on the extracellular membrane and within intracellular compartments of a limited number of cell types. In sheep with late stage classical scrapie, PrP^Sc ^is detected in neural tissue [3,4]. In preclinically infected sheep, PrP^Sc ^is detected in the lymphoreticular system [5] (including peripheral tissues such as nictitating membrane [6] and rectoanal mucosa-associated lymphoid tissue (RAMALT) [7] and in the placenta [8-10]. The infectious agent is found in blood [11] and milk [12], although generally at levels too low for reliable detection by conventional immunoassays [13]. Ovine scrapie is apparently transmitted by oral or mucosal exposure of lambs to the infectious agent shed in placenta, blood, colostrum or milk during the periparturient period [9,14].

Domestic goats (*Capra hircus*) are susceptible to ovine scrapie under field conditions [15] and a natural case of TSE with the biochemical characteristics of bovine spongiform encephalopathy (BSE) in a domestic goat has also been reported [16]. Under experimental conditions, goats are susceptible to several TSEs including ovine scrapie [17], cervid chronic wasting disease [18], human Creutzfeldt Jakob disease [19] and BSE [20,21]. Goats experimentally infected with BSE by the intracerebral route developed a TSE-like disease but failed to transmit disease to their progeny produced by embryo transfer or natural birth [21]. These reports did not address the potential for amplification and transmission of the agent should it occur under natural conditions. Although there are few reported cases of caprine scrapie in the United States, the introduction of antemortem diagnosis by peripheral lymphoid tissue biopsy has provided small numbers of naturally infected goats suitable for breeding. This study describes the sparse accumulation of PrP^Sc ^in shed placentas from the first 3 naturally infected breeding does identified by antemortem testing in the US and a 4^th ^infected doe with no antemortem evidence of scrapie.

## Results

### Antemortem and post-mortem scrapie diagnosis

Three domestic goats with exposure to ovine or caprine scrapie (1/3684C, 2/3953N, 2/3950 M, Table 1) were diagnosed with scrapie by antemortem detection of PrP^Sc ^in a biopsy sample of RAMALT using a monoclonal antibody-based immunohistochemistry (IHC) assay. A fourth goat (2/3987S) was negative by sequential RAMALT biopsy sample analysis at approximately 1, 1.5 and 2 years of age but held for quarantine because she was the progeny of a confirmed case. The 3 goats with recorded birthdates (2/3953N, 2/3950 M and 2/3987S) were euthanized with clinical signs of scrapie at 1041, 1146 and 906 days of age respectively. Goat 1/3684C developed clinical signs of scrapie at approximately 3-3.5 years of age. Clinical signs included weight loss, pruritis and incoordination. The diagnosis of scrapie was confirmed by post-mortem examination of the brainstem and lymphoid tissues by immunohistochemistry.

**Table 1:** Placentas from sheep and goats with naturally occurring scrapie: dam and fetal *PRNP *genotypes, disease status of dam

**Dam**				**Progeny**	
	
**Placenta** **ID^a^**	**Animal** **ID**	***PRNP^b^***	**Parturition to** **death (days)**	**Year born/** **No. progeny**	***PRNP***
	
G569	1/3684C	wt/wt	96	2006/1	wt/240P
	
G736A	2/3953N	240P/240P	332	2008/1	240P/240P
	
G736Z	2/3953N	240P/240P	332	2008/1	240P/146S240P
	
G742	2/3950M	wt/240P	431	2008/1	wt/240P
	
G797	2/3950M	wt/240P	83	2009/3	wt/240P
	
G836	2/3987S	240P/240P	181	2009/1	240P/240P
	
S345	2924	ARQ/ARQ	205	2005/3	ARQ/ARQ
	
S630	3345	ARQ/ARQ	249	2007/2	ARQ/ARQ
	
S738	3602	ARQ/ARQ	378	2008/2	ARQ/ARQ
	
S811	3602	ARQ/ARQ	22	2009/3	ARQ/ARQ
	
S758	3768	ARQ/ARQ	523	2008/1	ARQ/ARQ
	
S796	3768	ARQ/ARQ	181	2009/1	ARQ/ARQ
	
S850	4161	ARQ/ARQ	358	2009/2	ARQ/ARQ

^a ^Shed cotyledons from goats (G) or sheep (S)

^b ^PRNP genotypes included the wild type (wt) allele on a 240 S backbone with no other mutations, an allele differing only at codon 240 (240P) or an allele on the 240P backbone with a second polymorphism at codon 146 (S substituted for N) (146S240P)

### Parturition history

The 4 domestic goats held for observation because of a positive PrP^Sc ^finding on antemortem RAMALT assay (n = 3) or because of exposure to her infected dam at birth (n = 1) produced viable progeny and shed placentas. For comparison, five sheep with naturally acquired scrapie, originating from flocks unrelated to the infected goat herds, were used as a source of ovine placentas. *PRNP *genotypes, the number of progeny in each birth year, the *PRNP *genotypes of the progeny, and the interval from parturition to euthanasia of the dam with clinical scrapie are shown in Table 1. Caprine placentas were collected from 2 sequential pregnancies in one doe (2/3953N) and from the only live births for the other does. Ovine placentas were collected from single pregnancies (n = 3 sheep) or from 2 sequential pregnancies (n = 2 sheep).

#### PRNP genotypes of scrapie-positive goats and their progeny

The four scrapie infected breeding goats were homozygous for the wild type (wt) *PRNP *haplotype encoding 240 S (n = 1), homozygous for the alternative central haplotype encoding 240P (n = 2), or heterozygous for these two haplotypes (n = 1) (Table 1). *PRNP *genotypes of the kids included the heterozygous wt/240P genotype (n = 5), homozygous 240P (n = 2) and a single kid with the polymorphism at codon 146 encoding serine (240P/146S240P). Only one multiple birth resulted in mismatched alleles in placental cotyledons: in her only live kidding, goat 2/3953N produced one homozygous 240P kid and one kid heterozygous at codon 146 (wt/146S240P) (Table 1).

#### Immunoassays of placental tissue

##### Enzyme linked immunosorbent assay

ELISA testing was performed on shed cotyledons using the homogenate from 30 mg tissue per well (Table 2). All 27 ovine cotyledons (collected from 7 placentas) were positive by this assay, although the samples from one placenta (S850) were notably lower than those from the other placentas. In contrast, only 7/41 cotyledons collected from 2 of the 6 caprine placentas (or placental horns in the case of genetically mismatched fetuses G736A and G736Z) had PrP^Sc ^detectable with this assay. Two fold serial dilutions of caprine cotyledon homogenate (G569) showed that the assay was linear (r^2 ^= 0.97) over a range of 1.88 to 30 mg starting wet weight (data not shown). Based on this regression curve, the samples from the only other ELISA-positive caprine placenta (G797) and the weakly positive ovine placenta (S850) contained approximately 7 to 12 fold less PrP^Sc ^when compared to the reference caprine sample. All other ovine samples were positive for PrP^Sc ^in this assay with A_450 _values higher than that observed with any goat sample (Table 2). All other caprine samples were considered ELISA negative, below the cut-off value for the assay.

**Table 2:** Immunoassays for PrP^Sc ^in shed cotyledons from sheep and goats with naturally occurring scrapie

	Immunohistochemistry		ELISA		PTA-WB^a^
**Placenta**	**No. PrP^Sc^-pos cotyledons** **/no. tested**	**Relative abundance of PrP^Sc b^**	**No. PrP^Sc^-pos cotyledons** **/no. tested**	**mean A_450_^c^** **(SD)**	**No. PrP^Sc^-pos cotyledons** **/no. tested**

G569	8/9	+2	2/2	2.73 (0.23)	2/2

G736A	2/10	+1	0/7		6/6

G736Z	0/10	0	0/5		0/6

G742	11/11	+1	0/10		2/3

G797	3/10	+1	5/5	0.66 (.21)	7/7

G836	3/20	+1	0/12		1/6

S345	28/28	+1	4/4	2.82 (.27)	2/2

S630	2/2	+4	2/2	3.42 (.13)	2/2

S738	6/6	+4	3/3	3.44 (.18)	2/2

S811	6/6	+4	6/6	3.32 (.24)	2/2

S758	6/6	+4	3/3	3.08 (.54)	1/1

S796	3/3	+4	3/3	3.62 (.19)	4/4

S850	6/6	+1	6/6	0.43 (.07)	2/2

^a^Homogenates of individual cotyledons were precipitated with phosphotungstic acid and analyzed by western blot using a combination of mAb F99/97.6.1 and P4.

^b^One section each from the cotyledons with PrP^Sc ^immunolabeling was evaluated for the percentage of low power (10X) fields with PrP^Sc ^labelling and considered to have an abundance of 0 if no fields were positive, +1 if 10% or fewer of the fields were PrP^Sc ^positive, +2 if 11-25% were positive, +3 if 26-50% were positive, +4 if 51 = 75% were positive, and +5 if >76% were positive.

^c^Mean (SD) values for homogenates of cotyledons (number shown in the previous column), each tested in triplicate. Values below the cut-off limit for the assay are considered negative and not shown.

### Phosphotungstic acid-Western blot (PTA-WB) analysis

**Figure 1:**
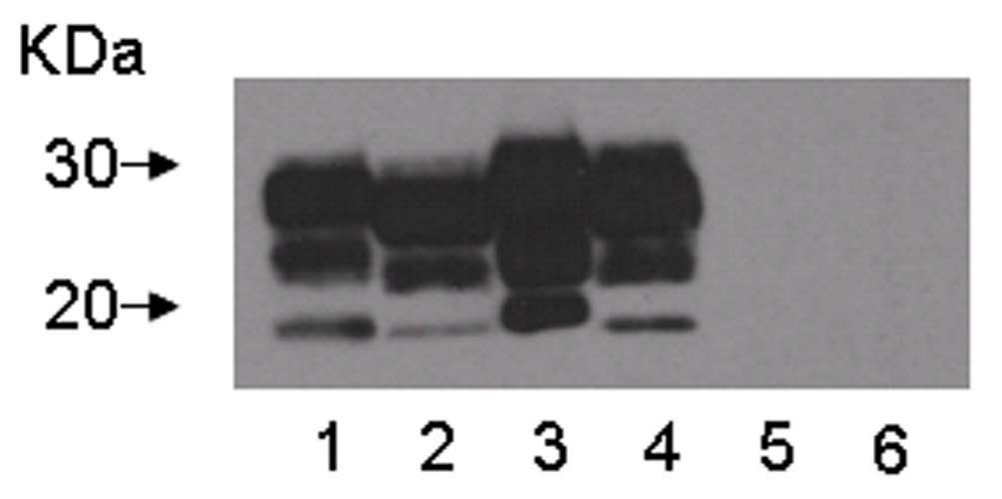
**Western blot analysis of brain and shed placental cotyledons from sheep and goats with naturally acquired scrapie**. Western blot detection of PrP^Sc ^using monoclonal antibodies F99/97.6.1 and P4 showing proteinase K resistant bands in samples of brain (lanes 1 and 3) and shed placental cotyledons (lanes 2 and 4) from goats (lanes 1 and 2) and sheep (lanes 3 and 4) with naturally acquired scrapie. Brain and placenta from a scrapie free goat (lanes 5 and 6) show no banding. Loading volumes were adjusted to produce similar banding intensity for comparison of migration patterns: positive and negative goat brain 3.2 mg per lane, positive sheep brain 0.8 mg, positive and negative goat placenta 150 mg, sheep placenta 30 mg. Arrows indicate molecular weight markers migrating at 30 and 20 kDa.

Paired samples of shed antemortem cotyledons and brain collected subsequently at necropsy from individual sheep and goats were prepared for western blot analysis following enrichment for PrP^Sc ^using PTA precipitation. Representative data are shown in Figure 1. Caprine and ovine brain (Figure 1, lanes 1 and 3) had a slightly reduced migration rate compared to caprine and ovine cotyledons (Figure 1, lanes 2 and 4) as reported previously for sheep [8]. Brain and placenta from scrapie negative goats showed no banding (Figure 1, lanes 5 and 6).

Using starting wet weight equivalents of 150 mg per lane, PrP^Sc ^was detected in all samples of ovine cotyledons examined by double antibody PTA-WB analysis. PrP^Sc ^was detected in 18/30 shed cotyledons from goats, with 4/5 placentas or placental horns containing at least one PrP^Sc ^positive cotyledon (Table 2). The only samples with no detectable PrP^Sc ^were the cotyledons from one horn of the uterus from the twin pregnancy of G2/3953N: 6/6 samples genotyped as 240P/240P were positive and 0/6 genotyped as 240P/146S240P were negative.

### Immunohistochemistry analysis of placental tissue

#### Shed cotyledons

**Figure 2:**
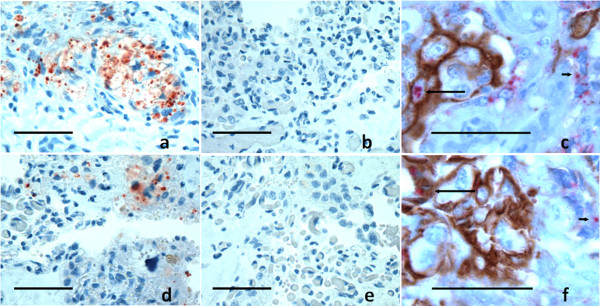
**Immunolabeling of shed and intact ovine and caprine placental tissue**. Single immunolabeling of PrP^Sc ^(red) in placentomes of scrapie-infected sheep (a) and goat (d) using mAb F99/97.6.1 and AEC immunolabeling, showing the reduction in PrP^Sc ^accumulation in caprine cotyledons relative to most ovine cotyledons. Sections from the same blocks labeled with an isotype control mAb show no labelling (b and e). Double immunolabeling using anti-prion antibody L42 (fuscia) and anti-pan cytokeratin, a marker of fetal trophoblasts (brown), demonstrates that PrP^Sc ^distribution in fetal trophoblasts (long arrows) and adjacent multinucleated cells (short arrows) was similar in the ovine (c) and caprine (f) placentomes. Photomicrographs were taken with an Olympus BX40 microscope coupled with an Olympus Q-Color3 camera. Axiovision software was used for scaling and Photoshop for formatting. Bar = 50 μm.

IHC was performed on single sections from each of multiple (n = 2-28 per placenta or placental horn) shed cotyledons from sheep and goats (Table 2). All sections of ovine cotyledon contained punctate and granular immunolabeling (Figure 2a) ranging from small scattered patches to areas in which most of the cells in each 10X field were considered PrP^Sc ^positive. Immunolabeled cells had the morphology of trophoblasts or remnants of multinucleated trophoblasts as well as fibrinoid material characteristic of placental villi. Immunolabeling characteristics in caprine cotyledons (Figure 2d) were similar to that seen in sheep, although the size of the immunolabeled plaques was generally smaller and more sparsely distributed. No immunolabeling was detected in placentomes from scrapie negative goats and sheep (data not shown) or from samples from positive goats or sheep in which the primary anti-PrP antibody was replaced by an isotype control antibody (Figure 2b and 2e).

All shed cotyledons from sheep had detectable immunolabeling (Table 2). Immunolabeling of caprine placentas was more variable, ranging from 90-100% of the sections examined in 2 placentas (G569 and G742) to 10-30% in the other 3 placentas). The only placenta or placental horn with no evidence of PrP^Sc ^detected by IHC in any of the shed cotyledons examined was from the 240P/146S240P twin (placenta G736Z) from goat 2/3953N; 0/10 cotyledons of this genotype were IHC positive and 2/10 cotyledons from the 240P/240P twin (placenta G736A) were positive.

Relative abundance of PrP^Sc ^in the shed cotyledons was estimated from the percentage of low power (10X) fields containing PrP^Sc ^IHC-immunolabeling (Table 2). Abundance of PrP^Sc ^detected by this method was variable in sheep: 2 samples were scored +1 (10% or fewer of the lower power 10X fields contained PrP^Sc^) and the remaining samples were considered +4 (50-75% of the fields contained PrP^Sc^). Caprine samples were uniformly low in abundance; one placenta was considered +2 (10-25% of the fields were positive) and the other sections with immunolabeling were considered +1.

#### Cellular distribution of PrP^Sc ^in intact placentomes

Cellular localization of PrP^Sc ^in the intact placentomes, containing both maternal and fetal tissue, was evaluated by IHC using placentomes collected at necropsy from goat 2/3953N at approximately 123 days gestation and placentomes from 4 scrapie infected sheep at a similar stage of pregnancy. PrP^Sc ^deposits were sparse in the caprine tissue but showed a distribution similar to that in ovine placenta. PrP^Sc ^immunolabeling was observed in uninucleated cells identified as trophoblasts by double immunolabeling with anti-pan cytokeratin and in adjacent multinucleated cells in the ovine (Figure 2c) and caprine (Figure 2f) placentomes.

## Discussion

Transmission of the ovine scrapie agent during or shortly after parturition was suggested by early studies [22] and evidence for the infectious agent and PrP^Sc ^in placental tissue is now well established [10,23,24]. PrP^Sc ^accumulation occurs early in pregnancy [8,25] and levels rise through the first 90-120 days of the 135 day ovine pregnancy. PrP^Sc ^is found in association with cells identified as uni- and multinucleated trophoblasts by morphology [8,9] and cytokeratin immunolabeling [9]. PrP^Sc ^immunolabeling is found occasionally in maternal caruncular endometrial cells, often in close contact with immunolabeled trophoblasts [8]. Although shedding of the agent into the lambing environment through placental tissue is a likely source of transmission, contamination of the maternal wool and udder by the dam's blood [11] may represent a risk for the newborn lamb in its early ineffectual search for colostrum. In addition, there is evidence for transmission by milk collected from some scrapie infected sheep [12,26-28]. Transmission of caprine scrapie is less well defined. Experimental transmission of ovine scrapie to goats by oral feeding with placenta was reported in early experimental trials [23] but the results of reciprocal experiments are not reported. Although several large scale studies of the distribution and characterization of PrP^Sc ^in goats have been described [29-31], placentas were not examined in those studies.

In this study, PrP^Sc ^was detected in all shed cotyledons examined from 7/7 ovine placentas using ELISA, IHC and PTA-WB assays. PrP^Sc ^was detected in at least one cotyledon from each of 5 caprine placentas. The percentage of PrP^Sc^-positive cotyledons and the relative abundance of PrP^Sc ^estimated by IHC or ELISA were generally lower in goats than in sheep, although considerable variation was observed in both species. There was some discordance among test formats. ELISA testing using a commercial kit manufactured for lymphoid tissue showed a slightly lower sensitivity than PTA-WB or IHC, possibly because PrP^Sc ^is distributed sparsely in the caprine tissue and may not have been present in the 30 mg samples tested in each well. PTA-WB has the advantage of a larger starting sample size (150 mg per lane) and detection with a combination of two antibodies (F99/97.6.1 and P4). Although IHC was performed with a single antibody (because reactivity of the P4 monoclonal antibody is abolished by formic acid pre-treatment used with these tissues), examination of a number of cotyledons was sufficient to identify positive placentas.

Only one set of caprine cotyledonary samples was negative on all assays; these samples were from a fetus heterozygous for the *PRNP *allele encoding 146 S on a 240P background. Placental accumulation of PrP^Sc ^in sheep is limited by the effect of fetal genotype [9,32,33] because the fetal trophoblast is a major site of PrP^Sc ^conversion or accumulation in the placenta. The highly resistant ovine *PRNP *allele encoding arginine (R) at residue 171 is associated with lack of detectable PrP^Sc ^in the placentomes of ovine fetuses [9,25,32]. Likewise, PrP^Sc ^was not detected in placentomes from fetuses with a dimorphism at *PRNP *codon 176, reported to confer resistance to classical scrapie, or at codon 141, associated with susceptibility to classical scrapie although with a prolonged incubation time [34]. The lack of PrP^Sc ^in the cotyledons with PRNP genotype 240P/146S240P is consistent with the field observations on relative resistance to scrapie in goats with this *PRNP *genotype [35,36]. Determining the effect of fetal genotype on PrP^Sc ^accumulation in the placentas of goats will be complicated by the large of number of caprine *PRNP *alleles on a dual wild type background [35-38].

The effect of the origin of the scrapie agent remains to be determined. In this study, herd 1 co-mingled scrapie infected sheep with goats. In herd 2, sheep had not been housed with goats for several years before the index case was diagnosed and therefore direct sheep to goat transmission is an unlikely source of the agent in this herd. Biochemical or bioassay tests to differentiate between scrapie of caprine or ovine origin will be useful in examining the effect of agent source on clinical outcome and transmission efficiency.

## Conclusions

In this study, PrP^Sc ^was detected in some but not all placentomes from naturally infected goats using a sensitive western blot assay. PrP^Sc ^detectable by IHC was sparsely distributed in caprine cotyledons and ELISA values were lower than observed with most ovine cotyledons. In spite of the poorly defined effects of *PRNP *genetics, scrapie strain, dose, route and source of infection, the caprine placenta may represent a source of infection to progeny and herd mates as well as a source of persistent environmental contamination. Caprine scrapie is rarely reported in the US and additional studies using experimentally infected goats may be useful in determining the role of the placenta in transmission of caprine scrapie.

## Methods

### Immunohistochemistry

Antemortem RAMALT tissues, necropsy samples, and placental tissues were immunolabeled using automated immunohistochemistry (IHC) as described [39] and modified [33]. Formalin fixed tissues were treated with 98% formic acid for one hour, rinsed and re-equilibrated in formalin before routine processing and paraffin embedding. Three μm sections were mounted on glass slides. Deparaffinization and antigen retrieval were performed online using proprietary reagents (Ventana Medical Systems, Tucson, AZ). Ovine RAMALT tissues were immunolabeled with a combination of mAb F89/160.1.5 (binding an epitope at residues 142-145) and mAb F99/97.6.1 (binding an epitope at residues 220-225) (VMRD, Pullman WA) at 2.5 μg/ml each, and detection with a peroxidase-labelled biotin-streptavidin system and amino-ethyl-carbazole as the chromogen (Ventana Medical Systems, Tucson, AZ USA). All caprine tissues (RAMALT and placental) and all ovine placental tissues were immunolabeled with mAb F99/97.6.1 alone at 5 μg/ml. Sections were counterstained with hematoxylin. Samples were considered positive for PrP^Sc ^if coarse bright red deposits were detected by bright-field microscopy. Positive and negative ovine lymphoid tissues were included in each run of 19 caprine and ovine placental tissues. Immunolabeling intensity of the positive control tissue was equivalent for all runs. Relative abundance of IHC immunolabeling in the shed cotyledons was graded on a scale of 0-5 by determination of the number of low power (10X) fields containing PrP^Sc^; 0 = no labelling; +1, 10% or fewer of the 10X fields positive; +2, >10% - 25% of 10X fields positive, +3, >25%-50% of 10X fields positive; +4, >50%-75% of fields positive; +5, >75% of fields positive. Ovine and caprine sections used to determine relative abundance were immunolabeled in the same run to reduce interassay variability.

Double immunolabeling was performed with an anti-pan cytokeratin primary monoclonal antibody (AE1/AE3) (Ventana Medical Systems, Tucson AZ USA) to detect fetal unicellular trophoblast cells [40]; the primary antibody was detected with a peroxidase labelled secondary antibody (goat anti-rabbit/anti-mouse) and 3,3' diaminobenzidine (DAB) (Ventana Medical Systems, Tucson, AZ USA) as the chromogen, followed by labelling with anti-prion mAb L42 recognizing an epitope at residues 145-163 (R-Biopharm AG, Marshall MI USA) at 0.21 μg/ml, and detection with an alkaline phosphatase labelled secondary antibody detected with Fast Red/Naphthol chromogen (Ventana Medical Systems, Tucson, AZ USA).

### Naturally infected goats and sheep

Naturally infected goats were identified in 2 herds, one with (herd 1) and one without (herd 2) known direct exposure to ovine scrapie. Herd 1 included approximately 100 mixed breed goats co-pastured with a flock of mixed breed, primarily white-faced sheep; scrapie was diagnosed in several sheep with clinical signs of weight loss, wool loss, or incoordination. At depopulation of the flock, 35% of the sheep were found to be infected. All infected sheep were homozygous for *PRNP *alleles encoding alanine at codon 136, arginine at codon 154 and glutamine at codon 171 (ARQ/ARQ). Live sheep from this herd were not available for research studies. Herd 2 included approximately 60 purebred Alpine, Nubian, and Angora goats. No sheep had been housed in this facility for the preceding five years and the founder goat population was increased by births and by purchased animals. Scrapie was diagnosed by post mortem analysis of brain and lymphoid tissues in one clinically suspect goat (designated the index case).

Naturally infected sheep for this study were donated by the US Department of Agriculture, Animal Plant Health Inspection Service following diagnosis of scrapie in one or more animals in the flock of origin or were progeny of infected ewes born in the quarantine facility. These sheep were unrelated to the infected animals in herd 1.

### Antemortem diagnosis

In herd 1, 100 goats were tested by RAMALT biopsy analysis. Two goats with PrP^Sc^-positive results were identified and transferred to the USDA quarantine facility in Pullman WA, USA. One goat kidded at approximately 2.5 years of age and was euthanized with clinical signs of scrapie 96 days later (Table 1). The other goat remains clinically normal and been excluded from this study because scrapie has not been confirmed by analysis of brain. The remaining goats from herd 1 were retained under permanent quarantine by the producer and were not available for follow-up testing. All goats from herd 2 were submitted to the quarantine facility following diagnosis of scrapie in the index case, a 3 year old Nubian goat. PrP^Sc ^was detected in 2/55 goats by RAMALT testing. In addition, one yearling goat (2/3987S) had no detectable PrP^Sc ^in the RAMALT sample but was held at the quarantine facility for breeding because she was the progeny of the index case. The other goats from herd 2 were submitted for post-mortem necropsy analysis. Three of these goats had PrP^Sc ^detectable in the brain; 2 of the 3 had no detectable PrP^Sc ^in lymphoid tissues.

### Clinical disease and post-mortem diagnosis

Goats and sheep were observed daily for clinical signs suggestive of scrapie, including pruritis, weight loss, and incoordination. Animals with advanced clinical disease were euthanized by intravenous overdose of sodium pentobarbital. At post-mortem, a tissue panel including brain, palatine tonsil, medial retropharyngeal lymph node and placenta (if pregnant) was collected. Tissues were immediately fixed in 10% neutral buffered formalin or frozen and stored at -20°C or -80°C. All animal procedures were approved by the Washington State University Institutional Animal Care and Use committee.

### Collection of shed cotyledons and intact placentomes

Shed placental tissues were collected from naturally infected sheep (n = 5 sheep, 7 pregnancies) and goats (n = 4 goats, 5 pregnancies) (Table 1) at parturition. Adult goats arrived at the facility in mid-gestation and/or were bred in subsequent years by natural service. Goats were held in paddocks that had previously housed scrapie-positive sheep but were not given direct exposure to sheep after arrival. Goat 1/3684C kidded in the paddock. All other pregnant scrapie-infected or exposed goats were transferred to animal biosafety level 2 facilities with concrete flooring 2-4 weeks before kidding. No scrapie infected sheep had been held in these buildings for a year prior to introduction of the goats. Pens were bedded with 3-6 inches of pine shavings. All goat kiddings were attended and placentas collected immediately from the layer of shavings and processed within one hour of shedding. Sheep were housed in paddocks and transferred to cement floored isolation buildings for parturition. Placentas were collected immediately after lambing. Ovine and caprine fetal cotyledons were processed by fixation in 10% neutral buffered formalin or frozen and stored at -20C.

Intact placentomes were collected at necropsy from goat 2/3953N after euthanasia due to clinical signs of scrapie at 123 days of gestation. Intact placentomes were collected from 4 domestic sheep with scrapie at approximately 120 days gestation.

### PRNP genotyping

The diploid *PRNP *genotype of all goats was determined by DNA sequence analysis. Haplotypes were assigned as described in our earlier study [37]. There is no standard nomenclature for caprine *PRNP *haplotypes. By convention, *PRNP *genotypes and haplotypes are described by the deduced amino acid residue at polymorphic sites under investigation. At least 25 coding changes in the open reading frame of the caprine *PRNP *gene have been described [41]. In contrast to the single ovine *PRNP *wild type allele, the *PRNP *polymorphisms in goats are found on two central haplotypes differing only at codon 240. The allele encoding 240 S is considered the caprine wild type (wt) allele in this study. None of the other reported polymorphisms on the 240 S background were found in the animals in this study. The second background allele differs encodes a substitution of proline (P) for S and is designated 240P in this study. Two additional haplotypes on the 240P background found in this group of goats included the substitution of S for asparagine at residue 146 (146S240P) and substitution of S for glycine at residue 127 (127S240P). In the case of the single pregnancy with *PRNP *mismatched progeny, *PRNP *genotypes of individual cotyledons were determined before immunoassay and the results are shown separately for progeny and cotyledons of each genotype in Tables 1 and 2.

### PrP^Sc ^detection in shed cotyledons and intact placentomes

#### ELISA

Shed fetal cotyledons were analyzed for PrP^Sc ^using a commercially available enzyme linked immunosorbent assay (ELISA) kit developed for detection of abnormal PrP in the lymphoid tissues of deer (IDEXX Laboratories, Westbrook ME USA); the kit has been used for assay of ovine placental tissue [33] although the kit has not been validated for use with this tissue. Each cotyledon was prepared as a 30% homogenate, with 100 μl (equivalent to 30 mg starting wet weight) loaded in each well; homogenates were tested in triplicate. Positive and negative controls provided with the kit were included in each run. The cut-off value for a positive result was calculated as the mean of duplicate negative control wells + 0.150. Samples with mean A_450 _values less than the cut-off value were considered negative. The mean and standard deviation of A_450 _values were calculated for cotyledons from each placenta (if the fetal genotypes were identical) or uterine horn (for the single placenta with *PRNP *mismatched twins). The dynamic range of the assay was determined using duplicate wells of 9 serial 2-fold dilutions of homogenate from caprine placenta G569, with final wet weight equivalents corresponding to 58 μg to 30 mg per well.

#### PTA-Western blot

Shed fetal cotyledons and cotyledons prepared from placentomes collected at necropsy were analyzed by PTA enriched western blot analysis as described [33]. Briefly, 30% (w/v) homogenates were treated with sodium phosphotungstic acid [42] to precipitate PrP^Sc^; precipitates from samples equivalent to 150 mg starting wet weight of tissue were treated with proteinase K (50 μg/ml final concentration, 37C, 1 h) and analyzed by western blot analysis using primary mAb F99/97.6.1 (VMRD Inc, Pullman WA USA) combined with mAb P4 (binding an epitope at residues 93-99) (R-Biopharm AG, Marshall, MI USA).

## Authors' contributions

KO designed the experiment, collated the experimental data and drafted the manuscript. DZ performed the ELISA testing and analyzed those data, as well as performing the Western blot analysis. DS performed the post mortem analysis and helped draft the manuscript. TT developed the double immunostaining method. TT and HY performed the immunohistochemistry assays and analyzed those data. All authors have read and approved the final manuscript.
